# Two Cases of “Throat Deafness,” with Some Observations on the Same, and a New Method of Application for Its Relief

**Published:** 1859-11

**Authors:** Frederic D. Lente

**Affiliations:** Cold Spring, N. Y.


*Two Cases of "Throat Deafness," with some Observations on the same, and a New Method of Application for its Relief.* By FREDERIC D. LENTE, M. D., of Cold Spring, N. Y.

CASE I. Miss Emma O., aged 18, a teacher in one of the "ward schools" of the city of New York, applied to me, June 26th, 1859, complaining of deafness, which has been gradually increasing for some months, until it has almost incapacitated her from pursuing her occupation. She states that, for some weeks past, while chewing at her meals, the deafness is complete, so that although she sees that a person at table is addressing her, she hears no sound whatever. She has also considerable irritation about the larynx, and an accumulation of adhesive mucus there in the morning; also a feeling of "weakness" at the top of the *sternum*. She considers her general health good, although she has rather a delicate appearance. Appetite and digestion good; bowels and catamenia regular. No evidence of disease of chest upon physical exploration. The *meatus auditorius externus* and *membrana tympani* have a normal aspect; the mucous membrane of the fauces also has a tolerably healthy appearance, and there is no hypertrophy of the tonsils. The ticking of a watch is heard at the distance of four inches from the left ear, and about half an inch from the right with some difficulty. Hears no noises in the ears, and has had no pain there. Asked the advice of a professor in one of the medical colleges of the city, who applied nitrate of silver to the throat with a probang, with some relief to the throat symptoms.

*Treatment.*—Not to go into a tedious detail of the daily treatment, it is sufficient to state that a solution of the nitrate of silver was applied to the throat four times, at an interval of three or four days, with relief to the symptoms referable to that part. She was directed to keep up a moderate pustulation over the upper part of the sternum and under the clavicles, with croton oil liniment; and a solution of the iodide of zinc in equal parts of glycerine and water was applied directly to the orifice of the Eustachian tube by the following method: The nozzle of a small glass syringe was inserted into the open end of a piece of elastic catheter (No. 4 or 5), and secured by a wrapping of thread. A small portion, some twenty or thirty drops, having been drawn into the syringe, the catheter was introduced into the nostril, with the lower *fenestra* looking outwards, and pushed backward to the posterior wall of the pharynx, about three inches and a quarter, the *fenestra* being then just opposite the opening of the Eustachian tube;* and upon using the injection with some force, the fluid was, of course, thrown directly upon it. A smarting sensation, extending to some little distance down the throat, and forwards towards the nose, was felt for an hour or two, but was not complained of. The strength of the solution was five grains to the ounce, increased to ten or more as the parts became habituated to its use. The application was made daily (on alternate days to either side), until the 14th of July, with an interruption for four or five days during the patient's temporary absence in the city; after which it was continued

* An English (Hutchinson's) catheter is preferable, as this has only one *fenestra*, and this in a favorable position for our purpose.

every other day for ten days longer, with the following result: At the expiration of a week, there was very sensible improvement in the hearing of the left ear, and at the end of another week, the hearing on this side was normal, and the application to it discontinued. The improvement of the right ear was more gradual, but still progressive; and within four weeks the hearing on this side was also perfectly restored, and as good during mastication as at any other time. Patient was also kept on the use of Garnier and Lamoureux's *dragées* of the citrate of iron, six grains per day, with considerable improvement of her general health and strength. She is now, several weeks after the discontinuance of the treatment, apparently well in every respect.

CASE II. Mrs. L., aged 40, general health good, applied to me August 12th, 1859. Says that, for some weeks past, she has had a "fullness and pain" in the region of the lachrymal sac and left nasal passage, also an increasing deafness in the left ear. She says the ear frequently, during the day, appears to be "stopped up," and it is at the same time the seat of a buzzing noise; but that, on *bowing the head forward* for a short time, the obstruction seems to be temporarily removed, and the buzzing ceases. The *meatus auditorius* and *membrana tympani*, as well as the fauces and tonsils, appear healthy.

*Treatment.*—Applied a leech to the Schneiderian membrane on the left side, which was followed by a considerable flow of blood, and relieved the nasal symptoms considerably; but the deafness remained the same. Used the injection of the *iodide of zinc*, as in the last case, with considerable relief to the deafness after the first application, and with entire relief after the third; the application having been made every other day.

The two cases just described will sufficiently indicate the nature of the deafness which it is proposed to treat by a simple application to the orifice of the Eustachian tube, and the manner of making this application. The particular kind of injection is perhaps not a matter of much importance. I was first induced to use the *iodide of zinc* from the fact that it has been sometimes found efficacious in making a favorable impression on chronic engorgement and hypertrophy of the tonsils, a form of disease usually very intractable under all other medical treatment, and because it is much less disagreeable and persistent to the taste than *nitrate of silver*, and does not stain the clothes or the skin so indelibly.

It was at one time extremely fashionable among aurists to attack the Eustachian tube, and through it, the *cavitas tympani*, for the cure of various forms of deafness, by means of sounds, catheters, medicated air douches, and injections; but these applications were eventually found to be more injurious than useful in a majority of cases, and they have been rapidly falling into disrepute. It has not, however, been customary to make the application simply to the *orifice* of the tube, at least in an efficient manner, while it is here that "throat deafness," and deafness arising from disease of the *cavitas tympani*, without doubt, as a general rule, have their origin. Disease of the Eustachian tube, at any other part than near its faucial opening, is one of the rarest affections of the ear, as shown by actual statistics (*see* Toynbee and Wilde); therefore, it can seldom be requisite, even if the attempt were devoid of danger, to make applications throughout its whole extent, especially as the effect of a stimulant on a mucous canal extends considerably farther than the point of application. When, on the other hand, we call to mind the numerous affections of the fauces which prevail in almost all climates, but especially in ours, both from common and specific causes, but more particularly from the phthisical and strumous diathesis, and from constant exposure to the ever-varying temperature and hygrometrical state of the atmosphere, we cannot wonder that the Eustachian tube, always influenced by these affections, should be frequently attacked with chronic disease. Both Toynbee and Wilde, our best authorities, agree that chronic disease of the cavity of the tympanum is by far the most common cause of deafness. And it is not at all improbable that this disease (chronic inflammation generally) is the result of the extension of repeated attacks of inflammation of the faucial extremity of the Eustachian tube; which, if recognized, and efficiently treated in time, might perhaps generally be arrested. The most intractable form of inflammation of the ear, which results from *scarlatina* no doubt, as Mr. Wilde believes, is the result of an extension of disease from the fauces along the tube; and this in all probability might be prevented by a timely and persevering application of appropriate remedies to the original seat of the disease, the extremity of the tube. So, after *measles* also, especially in strumous subjects, we should always be on the watch for this difficulty.

Even in the time of Hippocrates, as Mr. Wilde informs us, it was remarked that "quinsy" was followed by closure of the Eustachian tube, and consequent deafness. But, unfortunately, this closure was then, as it is most generally now, regarded as the result of the enlargement of the tonsil, and remedies consequently addressed to this part. Mr. Wilde most strenuously contends against considering deafness as dependent on tonsillitic enlargement; and probably with good reason, for it does seem almost impossible that an ordinary hypertrophy of the tonsil, however great, should extend so far upward and backward as to encroach upon the orifice of the tube. But in cases of tonsillitis, the inflammation, of course, extends to the adjacent parts, for instance, to the Eustachian tube, which is near by, and eventually tends just as effectually to produce its closure, more or less complete, by inflammatory exudation, as if the enlarged tonsil itself pressed upon it. This may satisfactorily explain the discrepancy of opinion among aurists as to the cause of "throat deafness." But the ablation of the tonsil has not unfrequently, according to the statements of reliable aurists, produced an almost immediately beneficial effect on the deafness. And why not? The hypertrophied body, three or four times its normal size, has kept up by its irritation a state of chronic inflammation of the adjacent mucous membrane, and its removal is not unnaturally followed by a favorable change in the condition of this membrane.

The uncertainty of all treatment addressed to chronic disease of the ear, and especially to disease of the Eustachian tube and cavity of the tympanum, the danger attending most of the methods of treatment recommended, even in skillful hands, must be my excuse for giving such prominence to the two cases detailed in this paper, cases possessing, *per se*, so little novelty or interest.—*American Journal of the Medical Sciences.*

